# APP family member dimeric complexes are formed predominantly in synaptic compartments

**DOI:** 10.1186/s13578-023-01092-6

**Published:** 2023-08-02

**Authors:** Sandra Schilling, Alexander August, Mathieu Meleux, Carolin Conradt, Luisa M. Tremmel, Sandra Teigler, Virginie Adam, Ulrike C. Müller, Edward H. Koo, Stefan Kins, Simone Eggert

**Affiliations:** 1 Department of Human Biology and Human Genetics, University of Kaiserslautern, 67663 Kaiserslautern, Germany; 2 Present Address: Department of Neurogenetics, Max-Planck-Institute for Multidisciplinary Sciences, City-Campus, Hermann-Rein-Str. 3, 37075 Göttingen, Germany; 3 Department of Neuroscience, University of California, San Diego (UCSD), La Jolla, CA 92093-0662 USA; 4 Present Address: Medical, Biochemistry & Molecular Biology, Center for Molecular Signaling (PZMS), Saarland University, 66421 Homburg, Germany; 5 Institute for Pharmacy and Molecular Biotechnology, University of Heidelberg, 69120 Heidelberg, Germany

**Keywords:** Alzheimer’s disease, Amyloid precursor protein, APLP1, APLP2, Dimerization, Synaptosomes, Blue Native gels

## Abstract

**Background:**

The amyloid precursor protein (APP), a key player in Alzheimer’s disease (AD), is part of a larger gene family, including the APP like proteins APLP1 and APLP2. They share similar structures, form homo- and heterotypic dimers and exhibit overlapping functions.

**Results:**

We investigated complex formation of the APP family members via two inducible dimerization systems, the FKBP-rapamycin based dimerization as well as cysteine induced dimerization, combined with co-immunoprecipitations and Blue Native (BN) gel analyses. Within the APP family, APLP1 shows the highest degree of dimerization and high molecular weight (HMW) complex formation. Interestingly, only about 20% of APP is dimerized in cultured cells whereas up to 50% of APP is dimerized in mouse brains, independent of age and splice forms. Furthermore, we could show that dimerized APP originates mostly from neurons and is enriched in synaptosomes. Finally, BN gel analysis of human cortex samples shows a significant decrease of APP dimers in AD patients compared to controls.

**Conclusions:**

Together, we suggest that loss of full-length APP dimers might correlate with loss of synapses in the process of AD.

**Supplementary Information:**

The online version contains supplementary material available at 10.1186/s13578-023-01092-6.

## Background

The amyloid precursor protein (APP) plays an essential role in Alzheimer’s disease (AD), since sequential cleavages of β- and γ-secretase lead to the formation of the 4 kDa Aβ peptide, which accumulates in brains of AD patients [1]. APP is part of a larger gene family, which includes two mammalian homologues, the amyloid precursor like protein 1 and 2 (APLP1 and APLP2) [2]. The APP family members share several common features like neuronal localization including prefrontal cortex and processing by α-, β-, and γ-secretase [3–7]. Mice with single and combined gene knockouts of the APP family show impairments in spatial learning [8–11], long-term potentiation [8–14], decreased number of dendritic spines [9, 10, 12, 15, 16] and profound neuromuscular junction (NMJ) defects [17–21]. Thus, loss of the physiological function of APP/APLPs might contribute to the loss of synapses in AD [22]. This is further corroborated by two recent studies of conditional APP/APLP1/APLP2 triple KO mice, in which APP family members were deleted in excitatory forebrain neurons either during development [23] or in the postnatal forebrain [24]. Postnatal gene deletion using CamKII-Cre mice resulted in impairments in hippocampal synaptic plasticity, learning and memory as well as neuronal hyperexcitability [24]. Gene deletion during embryonic development using Nex-Cre mice [23] showed an even more pronounced phenotype with aberrant hippocampal lamination, reduced dendritic length and spine density, severely impaired LTP and completely disrupted learning [23].

APP/APLPs also share a common domain structure [25]. Their large ectodomains include the so called E1 and E2 domains [5]. It has been shown that APP, APLP1, and APLP2 can dimerize in a homotypic and heterotypic manner [15, 17, 26]. Thereby, the E1 domain has been identified as the main interface of *cis*- as well as *trans*-cellular dimerization [26–28]. So far, different physiological functions have been implied for *cis*- and *trans*-dimerization of APP/APLPs. It was shown that *cis*-homodimerization of APP [29], as well as *cis*-heterodimerization of APP with the APP homologues APLP1 or APLP2 [28], decreases Aβ production. APP *cis*-dimerization leads to an altered subcellular localization presumably via interaction changes with LRP1 and differences in retrograde transport with SorLA, two known risk factors in AD [30–32]. In line with those results, a report from Willnow and colleagues showed a strong increase of APP dimers in brains of SorLA KO mice, underlining an interplay of the AD risk factor SorLA and APP dimerization [33].

Significantly, *trans*-dimerization of APP proteins has been reported to promote cell–cell adhesion [15, 17, 26, 34]. This is supporting the notion that APP and its homologues might function as synaptic cell adhesion proteins [15]. Therefore, we aimed to investigate APP/APLP dimerization in brain in more detail, in this study, using two different in vitro dimerization assays, based on cysteine mutations at putative interaction sites and the FKBP-rapamycin dimerizer system.

## Results

### Characterization of APP family dimer complexes via the FKBP-rapamycine system

APP and its mammalian homologues are known to dimerize in *cis*- as well as in *trans*-orientation [26]. In this study, we want to investigate complex formation of all APP family members via BN gel analyses via two inducible dimerization systems which we already established for APP, firstly the FKBP-rapamycin system and secondly via constitutive cysteine-induced dimerization [29, 30].

**Fig. 1:**
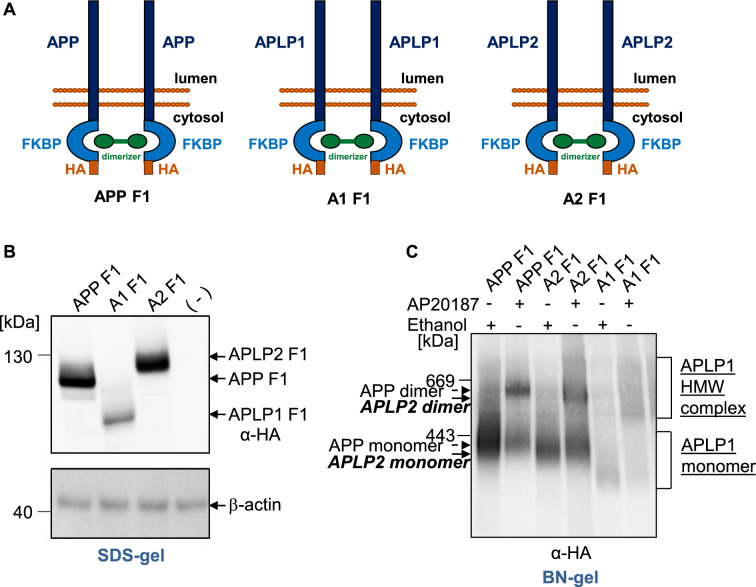
Comparative analysis of FKBP induced dimerization of the APP gene family members. **A** Schematic illustration of the used constructs APP F1, APLP1 F1 or APLP2 F1. By addition of the rapamycin analogue AP20187 the FKBP tags can be dimerized. **B** The FKBP tagged APP gene family members were heterologously expressed in N2a cells. Non-transfected N2a cells served as a negative control. Equal amounts of cell lysates were separated on denaturating SDS-Gels and Western blot detection followed with an α-HA antibody. Detection with primary antibody β-actin was performed as a loading control. **C** APP F1, APLP1 F1 or APLP2 F1 were heterologously expressed in N2a cells and treated with 100 nM of the rapamycin analogue AP20187 overnight to induce dimerization of APP/APLPs. Incubation with the solvent of AP20187, ethanol, served as a negative control. The samples were analyzed via semi-denaturing Blue Native PAGE and Western blot analysis followed with an α-HA antibody

Therefore, a 12 kDa FKBP tag adjacent to an HA tag was fused to the C-terminus of APP_695_, APLP1, and APLP2_763_, termed APP F1, APLP1 F1, and APLP2 F1, respectively (Fig. 1A). Western Blot analysis under denaturing conditions of heterologously expressed fusion proteins in N2a cells, which had been lysed in buffer containing 1% NP40, revealed for APP F1, APLP1 F1, and APLP2 F1 an apparent molecular weight of ~ 130 kDa, 100 kDa and 140 kDa, as expected (Fig. 1B).

Addition of an analogue of rapamycin (which is cell permeable) to transiently transfected N2a cells should allow efficient dimerization of APP family members via binding two FKBP molecules [29, 30, 35]. N2a cells heterologously expressing APP F1, APLP1 F1 or APLP2 F1 were either treated with the vehicle ethanol as a control or with 100 nM of AP20180, the so called dimerizer, overnight. Cells were harvested in homogenization buffer without detergent and disrupted mechanically. Then, the complexes were solubilized out of the membrane using the mild detergent *N*-dodecyl-β-d-maltoside and separated on a gradient gel to detect complexes of APP, APLP1 or APLP2. For APP and APLP2, we visualized two distinct signals at 600–700 kDa and ~ 200–300 kDa (Fig. 1C). Similar to APP [29, 30], for APLP2, mainly two distinct signals with > 80% in the low molecular weight (LMW) complex and only a minor part, < 20%, in the high molecular weight (HMW) complex were detected in ethanol treated control cells. In contrast, APLP2 extracted from dimerizer treated cells showed about > 60% of APLP2 in the HMW complex. Quantification of the APP family monomers and dimers is shown in Fig. 5. Due to the almost identical apparent molecular weight compared with APP, these data indicate that the HMW signals of APLP2 represent dimeric APLP2, as shown for APP before [29, 30]. In contrast, APLP1 shows a different pattern compared to APP and APLP2 in the vehicle treated control with signals between 200 and 800 kDa, possibly representing monomeric and dimeric complexes between 200–400 kDa and 400–800 kDa, respectively (Fig. 1C).

### Characterization of APP family dimer complexes via newly introduced cysteines close to the transmembrane domain

**Fig. 2:**
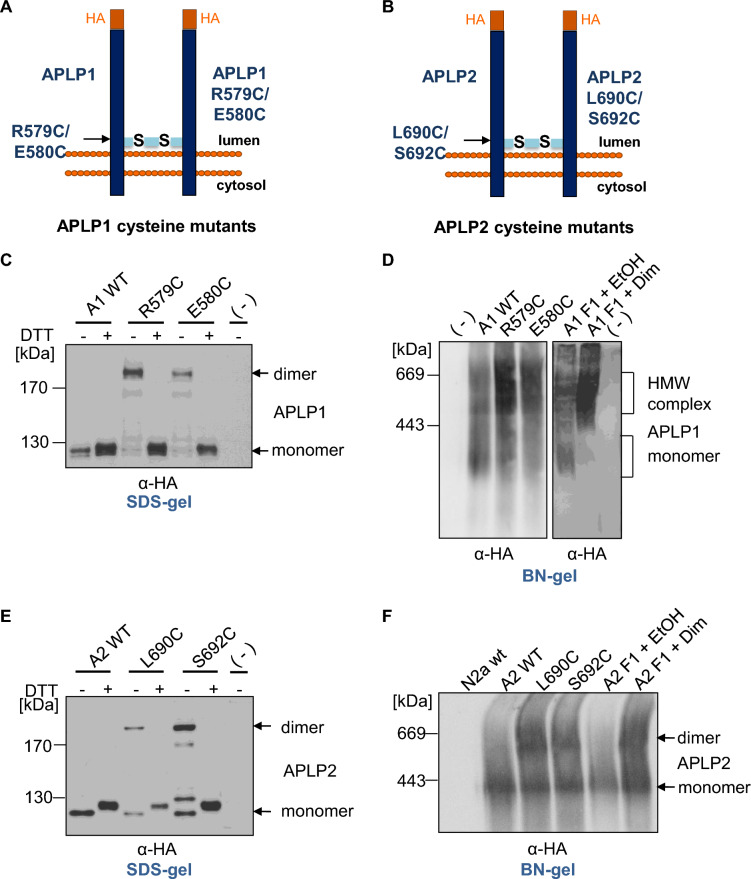
Comparative analysis of induced dimerization of APLP1 and APLP2 with cysteine mutants. **A**, **B** Schematic illustration of the used cysteine mutants for APLP1 and APLP2, **C** APLP1 WT, APLP1 R579C and APLP1 E580C were heterologously expressed in N2a cells. Non-transfected N2a cells served as a negative control. Equal amounts of cell lysates were prepared in samples with or without DTT, to allow visualization of cysteine dimers via disulfide bridges. Separation on denaturing SDS-gels followed and Western blot detection with an α-HA antibody. **D** APLP1 WT, APLP1 R579C, APLP1 E580C, and APLP1 F1 were heterologously expressed in N2a cells. APLP1 F1 expressing cells were either treated with 100 nM of the rapamycin analogue AP20187 overnight to induce dimerization of APLP1 or with the solvent ethanol as a negative control. Non transfected N2a cells served as a further negative control. The samples were analyzed via semi-denaturing Blue Native PAGE and Western blot analysis followed with an α-HA antibody. **E** APLP2 WT, APLP2 L690C, and APLP2 S692C were transiently transfected in N2a cells. Equal amounts of protein were denatured in sample buffer with and without DTT and analyzed after SDS PAGE via Western blot detection with α-HA antibody. **F** APLP2 WT, APLP2 L690C, APLP2 S692C, and APLP2 F1 were heterologously expressed in N2a cells. APLP2 F1 expressing cells were either treated with 100 nM of the rapamycin analogue AP20187 overnight to induce dimerization of APLP2 or with the same volume of the vehicle ethanol as a negative control. Non transfected N2a cells served as a further negative control. The samples were analyzed via Blue Native PAGE and Western blot analysis followed with an α-HA antibody

To corroborate our assumption that the HMW complexes represent dimeric forms of APLPs, we further analyzed cysteine induced APLP1 and APLP2 dimers, analogously to cysteine induced dimerization of APP, which we previously described using APP L17C and APP K28C containing amino acid substitutions within the Aβ domain [29, 30, 36, 37]. For this purpose, we were introducing cysteines N-terminally to the transmembrane domain of APLP1 and APLP2, assumed to cause formation of disulfide bridges between the two molecules containing the cysteine variants. We generated APLP1 containing the amino acid changes R597C and E580C (Fig. 2A) as well as the APLP2 variants L690C and S692C (Fig. 2B) using N-terminally HA tagged constructs and firstly examined APLP1 R597C and E580C via SDS gel electrophoresis compared to APLP1 WT. Non transfected N2a cells served as a negative control. We prepared cell lysates with or without DTT, allowing visualization of APLP1 covalent dimers on SDS gels, since disulfide bridges will remain intact in the absence of DTT. Western blot detection with an α-HA antibody revealed that for APLP1 R597C, a higher amount of APLP1 dimers was visualized compared to APLP1 E580C while for APLP1 WT almost no SDS resistant APLP1 dimers were present (Fig. 2C). This shows that APLP1 dimerization can be induced by incorporation of cysteines in their juxtamembraneous domains. Furthermore, these data indicate that dimer formation via covalent binding does not occur for WT APLP1.

We aimed to investigate the abundance of high molecular weight complexes of APLP1 via BN gel analyses. In order to assign the detected signals correctly to APLP1 monomers and dimers on BN gels, we compared induced dimer formation of APLP1 via cysteine mutants with induced dimer formation via the FKBP-rapamycin system on BN gels which revealed that APLP1 R597C and E580C show an accumulation of APLP1 high molecular weight complexes at about the same apparent molecular weight between 400 and 800 kDa as APLP1 F1 transfected cells treated with dimerizer overnight (Fig. 2D). Induced dimer/multimer formation was > 50% for both systems.

We performed the same kind of experiments for APLP2 and firstly prepared cell lysates of N2a cells expressing either APLP2 WT, APLP2 L690C or APLP2 S692C. SDS gel analysis followed by Western blot detection with an α-HA antibody revealed strong dimer formation for APLP2 S692C as well as APLP2 L690C in samples prepared using sample buffer without DTT. APLP2 WT did not show dimer formation under these conditions indicating that dimerization of APLP2 WT is not based on covalent interactions similarly as shown before for APLP1 WT (Fig. 2C, E). Next, we examined, analogously to APLP1, induced dimer formation of APLP2 via cysteine mutants in comparison to induced dimer formation via the FKBP-rapamycin system on BN gels. This indicated that APLP2 L690C and S692C show an accumulation of APLP2 dimers at the same apparent molecular weight as APLP2 F1 transfected cells treated with dimerizer overnight at ~ 600 kDa and monomeric APLP2 at ~ 300 kDa (Fig. 2F). Induction of APLP2 dimer formation was ~ 66% for both systems. Similarly, to APP WT and APP F1, APLP2 WT and APLP2 F1 showed only very little dimer formation of about 16% (Figs. 1C, 2F).

### Analysis of *cis*- and *trans*-dimerization of the APP family members via co-immunoprecipitations

**Fig. 3:**
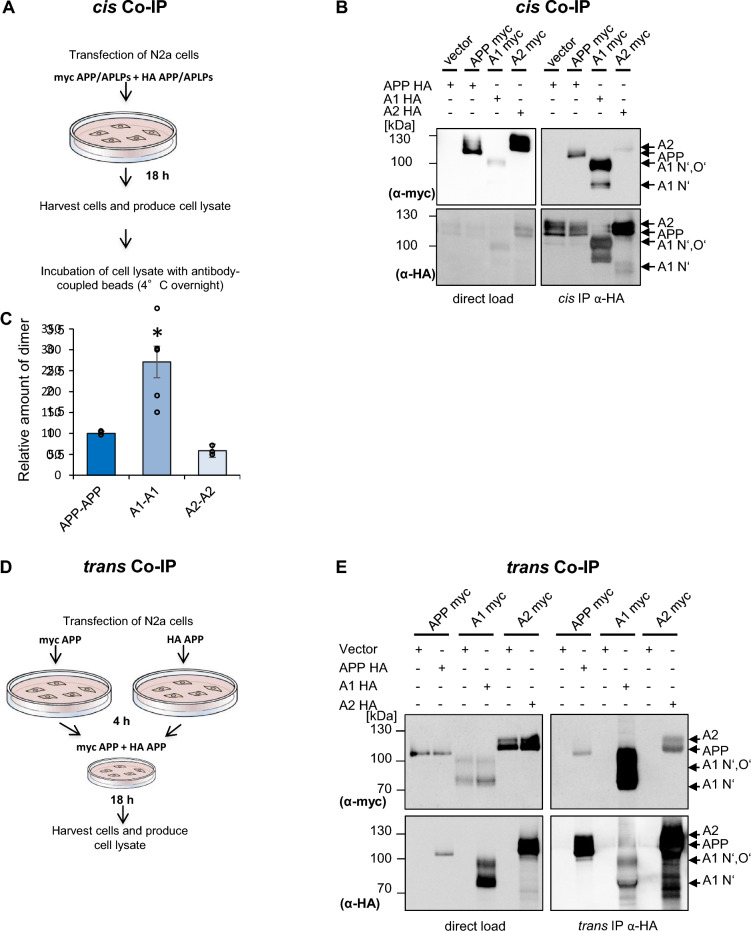
The extent of APP, APLP1, APLP2 *cis*-dimers and *trans*-dimers in N2a cells. **A** Experimental design of the *cis-*co-immunoprecipitation. **B** *Cis-*co-immunoprecipitation of APP, APLP1, and APLP2 (*cis*-homodimers). N2a cells were transiently co-transfected with APP c-myc and APP HA, APLP1 c-myc and APLP1 HA or APLP2 c-myc and APLP2 HA constructs. Co-transfection with an empty vector served as a negative control. Equal amounts of cell lysates were loaded on an SDS gel and analyzed via Western blot with primary α-HA or α-c-myc antibodies (input controls). Further, equal amounts of cell lysates were used for immunoprecipitation with α-HA coated agarose beads. The samples were separated on an SDS gel and subjected for Western blot detection with primary antibody α-c-myc to detect the co-immunoprecipitated proteins. The same membrane was incubated afterwards with α-HA antibody to visualize total amounts of immunoprecipitated proteins. **C** Quantification of data shown in A. Bars represent mean values ± SEM; *n* = 3, unpaired Student’s *t* test *p < 0.05, **p < 0.01, ***p < 0.001. **D** Experimental design of the *trans-*co-immunoprecipitation. **E** *Trans*-co-immunoprecipitation of APP, APLP1 or APLP2 (*trans*-homodimers). N2a cells were transiently transfected in separate dishes with APP, APLP1 or APLP2 HA or c-myc tagged constructs. Further, transfections of APP myc or empty vector served as a negative control. 4 h post-transfection, the transfected cells of two dishes were combined: APP myc and APP HA, APLP1 myc and APLP1 HA, APLP2 myc and APLP2 HA. Equal amounts of cell lysates were loaded on an SDS gel and analyzed via Western blot with primary α-HA or α-c-myc antibodies (input controls). Further, equal amounts of cell lysates were used for immunoprecipitation with α-HA antibody coated agarose beads. The samples were separated on an SDS gel and subjected for Western blot detection with the primary antibody α-c-myc to detect the co-immunoprecipitated proteins. The same membrane was incubated afterwards with an α-HA antibody to detect total amounts of immunoprecipitated proteins

Since we observed a markedly higher rate of APLP1 dimerization compared to APP and APLP2 via BN gel analysis, even without using a dimerization system, we wanted to confirm this result via a different method. Therefore, we analyzed the dimerization state of the APP family members via co-immunoprecipitation (Fig. 3A). N2a cells expressing c-myc and HA tagged APP, APLP1 or APLP2, respectively, were lysed 24 h post-transfection and subjected to co-immunoprecipitation with anti-HA antibody coupled beads. N2a cells co-transfected with vector and APP HA served as a control. Western blot detection with an α-c-myc antibody revealed almost comparable expression level of the c-myc tagged APP, APLP1, and APLP2 proteins with the lowest amount for APLP1 (Fig. 3B). In contrast, Western blot detection of the IP samples with an α-c-myc antibody demonstrated for APLP1 the strongest dimer signal of the APP family members, while APLP2 indicated the weakest homodimerization, which was confirmed by quantification of the integrated densities (Fig. 3C). Western blot detection of the same membrane with an α-HA antibody showed comparable signals of HA tagged APP/APLPs in the input control as well as of the immunoprecipitated proteins (Fig. 3B). Notably, the c-myc and HA tagged APP/APLP proteins were expressed in the same cells and thus the observed interaction likely took place in a lateral *cis*-directed fashion within the membrane. However, the interaction might also partially take place between APP/APLPs of neighbouring cells in a *trans*-cellular fashion.

To test more directly for *trans*-interaction of the APP family members, we modified the co-immunoprecipitation assay. N2a cells expressing either c-myc tagged APP or HA tagged APP were grown on separate cell dishes for 1 day post-transfection and then co-cultivated on one plate for one more day allowing formation of *trans*-cellular contacts (Fig. 3D). A small part of the cell lysates was loaded as an input control and the major part was used for the immunoprecipitation with HA beads. Western blot detection with an α-c-myc antibody revealed clearly that c-myc tagged APP can be co-immunoprecipitated with HA-tagged APP expressed in the neighbouring cells, indicating that APP can form *trans*-cellular dimers (Fig. 3E). Combination of N2a cells transfected with vector and c-myc tagged APP served as negative control. A post-lysis mixture control showed specificity of the co-immunoprecipitation system used (Additional file 1: Fig. S1).

Analogous experiments were also carried out with N2a cells expressing HA or c-myc tagged APLP1 or APLP2 on separate cell plates that were again co-cultivated for 1 day, lysed and subjected for co-immunoprecipitation. Combination of N2a cells transfected with vector and c-myc tagged APLP1 or APLP2 served again as negative controls. Interestingly, the highest amount of *trans*-interaction was detected for APLP1 (Fig. 3E). Taken together, we were able two show via *cis*- as well as via *trans-*co-immunoprecipitation that APLP1 has a considerably higher dimerization propensity in comparison to its homologues APP and APLP2.

**Fig. 4:**
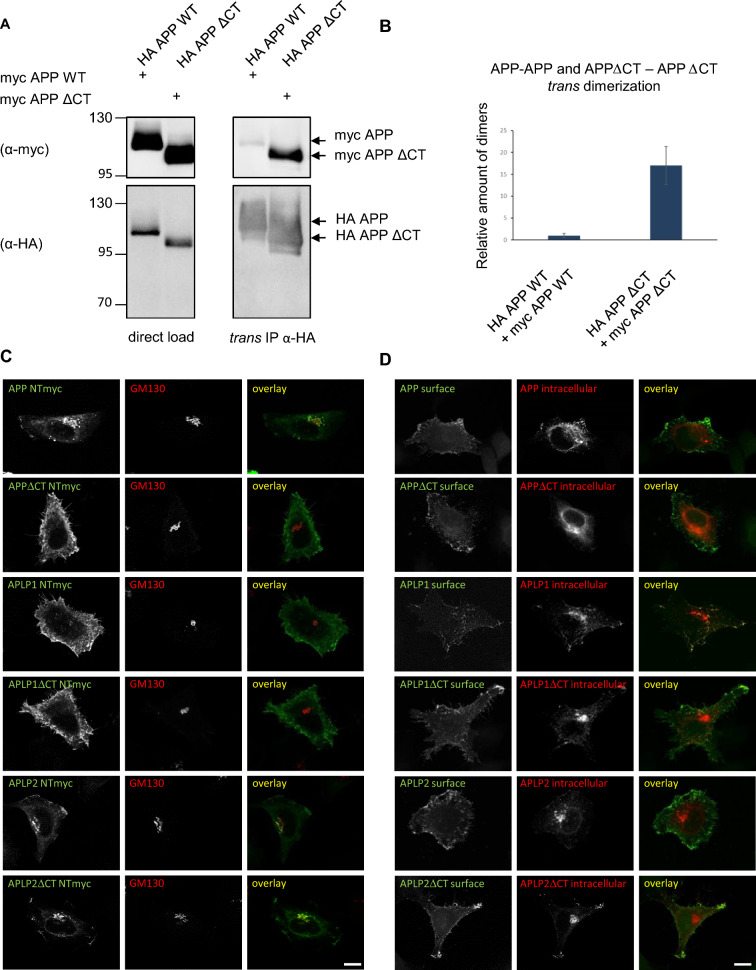
*Trans*-dimerization of the APP gene family members lacking the C-terminus. **A** *Trans*-co-immunoprecipitation of APP WT or APP∆CT, *trans*-homodimers. N2a cells were transiently transfected in separate dishes with myc APP, HA APP, myc APP∆CT or HA APP∆CT constructs. 4 h post-transfection, the following cells of separate dishes were combined: myc APP and HA APP, myc APP∆CT and HA APP∆CT. Equal amounts of cell lysates were loaded directly on an SDS gel and analyzed via Western blot with primary α-HA or α-c-myc antibodies (input controls). Further, equal amounts of cell lysates were used for immunoprecipitation with α-HA antibody coated agarose beads. The samples were separated on an SDS gel and subjected for Western blot detection with the primary antibody α-c-myc to detect the co-immunoprecipitated proteins. The same membrane was incubated afterwards with an α-HA antibody to detect total amounts of immunoprecipitated HA APP HA or HA APP∆CT. **B** Quantification of data shown in A. Bars represent mean values ± SEM; *n* = 3, unpaired Student’s *t* test *p < 0.05, **p < 0.01, ***p < 0.001. **C** HeLa cells were transiently transfected with N-terminally c-myc tagged APP, APP∆CT, APLP1, APLP1∆CT, APLP2 and APLP2∆CT. Cells were stained with α-c-myc antibody to visualize the overexpressed proteins and GM130 antibody to show the *cis*-Golgi apparatus in permeabilized cells. APP gene family members lacking the C-terminus are located to a higher amount at the cell surface. Scale bar, 10 µm. **D** Cell surface staining of HeLa cells transiently transfected with N-terminally c-myc tagged APP, APP∆CT, APLP1, APLP1∆CT, APLP2 or APLP2∆CT. Cells were incubated with an α-c-myc antibody on ice to stain only proteins which were localized at the surface. After fixation, the cells were permeabilized and stained again with an α-c-myc antibody to also visualize the intracellular proteins

Next, we addressed the question if the stronger presence of APLP1 *trans-*dimers may be due to higher APLP1 cell surface levels compared to the other APP family members. All APP family members are known to be internalized via clathrin-mediated endocytosis [15, 29] with the lowest internalization rate for APLP1, which therefore shows the highest presence at the cell surface [15]. To validate that higher cell surface localization of APP will also result in a higher amount of *trans*-dimers, we performed *trans*-co immunoprecipitations of APP∆CT lacking the entire C-terminus and therefore all endocytosis motifs and compared its propensity to form *trans-*dimers with APP WT (Fig. 4A, B). N-terminally HA tagged and N-terminally c-myc tagged APP∆CT constructs or N-terminally HA tagged and N-terminally c-myc tagged APP WT were transiently transfected into N2a cells. The differently tagged APP WT or APP∆CT expressing cells were combined 4 h after transfection and analyzed the following day. The HA tagged proteins were pulled down via HA beads and equal amounts of protein were loaded as input controls. Western blot detection of the input controls and IP samples with an anti-HA antibody confirmed that equal amounts of protein were loaded and that equal amounts of APP WT and APP∆CT were pulled down with HA beads (Fig. 4A). In contrast, detection with anti-c-myc antibodies demonstrated that onefold higher amounts of APP∆CT were pulled down via *trans*-co-immunoprecipitation compared with APP WT (Fig. 4B). This demonstrates that indeed, a higher presence of APP at the cell surface due to strongly impaired endocytosis [38] goes along with a higher amount of APP *trans*-dimers.

### Analysis of cell surface localization of the APP family members via immunocytochemistry

To further validate the impact of the C-terminus on cell surface localization of the APP family members, we performed an immunocytochemical staining with N-terminally c-myc tagged full length proteins and mutants lacking the entire C-terminus of all APP family members (APP WT, APLP1 WT, APLP2 WT, APP∆CT, APLP1∆CT, and APLP2∆CT). Firstly, we were analyzing localization of the APP family members in cells permeabilized with 0.1% NP40 using an anti-c-myc antibody and the *cis*-Golgi marker GM130 (Fig. 4C). APP WT showed a prominent Golgi and a vesicular staining with very little signal at the cell surface, while APP∆CT seemed to be present almost exclusively at the plasma membrane. For both APLP1 WT and APLP1∆CT, mostly cell surface staining and almost no co-localization with the *cis*-Golgi marker was observed. APLP1 WT showed also a punctate cytosolic localization, which was not present for APLP1∆CT (Fig. 4C). For APLP2 WT, a substantial presence in the *cis*-Golgi apparatus was detectable with almost no additional punctate staining, but a considerable presence at the cell surface, which was comparable to the staining pattern of APLP2∆CT (Fig. 4C). This suggests that lack of the APLP2 C-terminus including the internalization motifs surprisingly did not lead to a major shift to the presence at the cell surface.

Secondly, we were analyzing localization of the APP family members via cell surface staining (in non-permeabilized cells) and subsequent intracellular staining (permeabilized cells) using an α-c-myc antibody combined with two different secondary antibodies (surface: Alexa Fluor-488, *green*; intracellular: Alexa Fluor-594, *red*). The overlay indicates relative intensities of total (*red*) and cell surface (*green*) APP/APLPs (Fig. 4D). This set of immunocytochemical stainings clearly confirms that all APP family members are present at the cell surface. Taken together, we demonstrated in neuroblastoma cells that APLP1 shows the highest dimerization propensity of the APP family members, as well as the strongest presence at the cell surface.

### Analysis of APP family dimer complexes in WT mouse brains

**Fig. 5:**
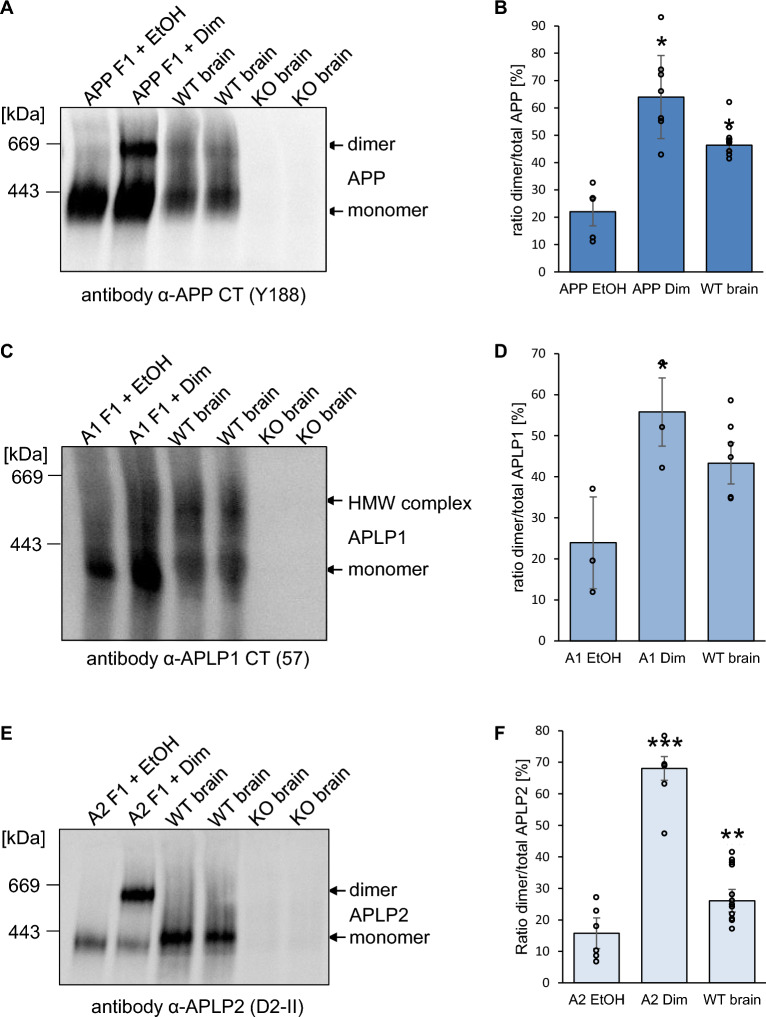
The extent of APP, APLP1, APLP2 dimers in N2a cells versus mouse brains. **A** APP F1 was heterologously expressed in N2a cells and treated either with 100 nM AP20187 over night to induce dimerization or with the same volume of ethanol as a control. One year old WT mouse cortices and APP KO mouse cortices were homogenized. All samples were prepared under semi-denaturing conditions for analysis on Blue Native gels. Western blot detection of APP followed with the primary α-APP C-terminal antibody Y188. **B** Quantification of data shown in A. Bars represent mean values ± SEM of the ratio dimer/total of FKBP-rapamycin induced APP dimer in N2a cells and endogenous APP dimer in mouse brains. *n* = 3. **C** APLP1 F1 was heterologously expressed in N2a cells and treated either with 100 nM AP20187 over night to induce dimerization or with ethanol as a control. In parallel, 1 year old WT mouse cortices and APLP1 KO mouse cortices were analyzed. Analysis of Blue Native gel samples was followed by Western blot detection of APLP1 with the primary α-APLP1 antibody 57. **D** Quantification of data shown in **C**. Bars represent mean values ± SEM of the ratio dimer/total of FKBP-rapamycin induced APLP1 dimer in N2a cells and endogenous APLP1 dimer in mouse brains. *n* = 3. **E** APLP2 F1 was heterologously expressed in N2a cells and treated either with 100 nM AP20187 over night to induce dimerization or with ethanol as a control. Those samples were compared to 1 year old WT mouse cortices and APLP2 KO mouse cortices. Analysis via Blue Native gels was followed by Western blot detection of APLP2 with the primary α-APLP2 antibody D2-II. **F** Quantification of data shown in **E**. Bars represent mean values ± SEM of the ratio dimer/total of FKBP-rapamycin induced APLP2 dimer in N2a cells and endogenous APLP2 dimer in mouse brains. *n* = 3

As a next step, we wanted to analyze complex formation of the APP family members in intact tissue. Therefore, we were comparing the FKBP fusion proteins of the APP family members heterologously expressed in N2a cells after induced dimerization with WT cortical mouse brain samples via BN gel analysis (Fig. 5). The corresponding KO brain samples served as a control to prove specificity of the antibodies used. APP F1 expressing cells treated with the vehicle control ethanol showed about 22.17% ± 4.7% APP dimers and ~ 65.8% ± 8.2% induced APP dimers, as expected [29]. In contrast, APP dimers in WT mouse brains were detected with an amount of ~ 48.9% ± 1.6%, a ~ 2× higher value compared to APP dimers in neuronal N2a cells (Fig. 5A, B). For APLP1 F1, about 31.8% ± 6.1% APLP1 dimers were detected in vehicle treated N2a cells while induced dimerization resulted in 55.8% ± 6.1% APLP1 dimers. In contrast, APLP1 in cortical mouse brains was dimerized 45.6% ± 3.9%, similarly as APP (Fig. 5C, D). APLP2 F1 expressing cells treated with the vehicle control ethanol showed about 16.24% ± 3.4% APLP2 dimers and 66.15% ± 4.3% induced APLP2 dimers, similarly as for APP F1. In contrast, much less APLP2 dimers were identified in WT mouse brains compared with APP and APLP1 with amounts to only 26.07% ± 3.60% (Fig. 5E, F). Taken together, dimerization of APP in mouse brains was strongly increased compared to N2a cells while dimerization of APLP1 and APLP2 was comparable.

**Fig. 6:**
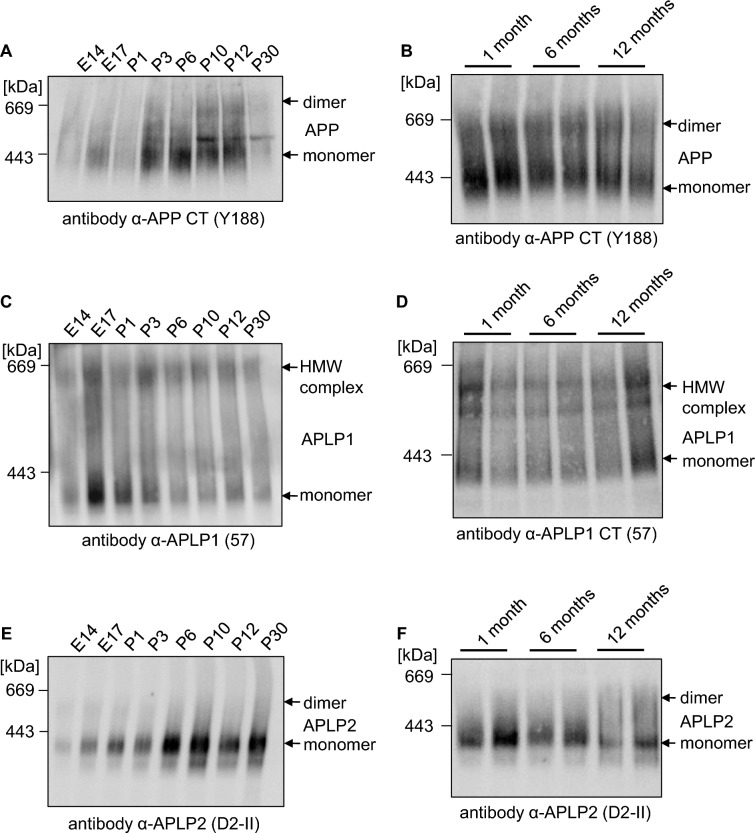
Dimer amount of the APP gene family members differs during development. **A**–**F** Protein expression analysis of dimers of the APP gene family members in mouse cortices during development. Mouse cortices of developmental stages E14, E17, P1, P3, P6, P10, P12, P30, 1 month, 6 months, and 12 months were lysed. All samples were prepared under semi-denaturing conditions for analysis on Blue Native gels. Western blot detection of APP followed with the primary α-APP C-terminal antibody Y188 (**A**, **B**), of APLP1 with the primary α-APLP1 antibody 57 (**C**, **D**) and APLP2 with the primary α-APLP2 antibody D2-II (**E**, **F**)

As a next step, we wanted to analyze complex formation of the APP family members in cortical mouse tissue in more detail. Therefore, we were examining dimer formation at different developmental time points, E14 (embryonic day 14 of a mouse pregnancy), E17, P1 (postnatal, P3, P6, P10; P12, and P30 (Fig. 6A, C, E).

We also assessed the abundance of APP, APLP1, and APLP2 dimers during adulthood at 1, 6, and 12 months of age (Fig. 6B, D, F). APP was visualized with the KO verified specific antibody α-Y188 and increasing APP complex formation was detected with little dimer formation starting E14 and the peak at P10/P12 (Fig. 6A).

APP expression during different developmental stages, E14, E17, P3, P6, P10, P12, P30, P65 has already been analyzed via SDS-PAGE and confirms the upregulation in APP protein expression P3–P12 [15].

The amount of APP dimers was unchanged between 1 and 12 months old mice and constantly ~ 49% (Fig. 6B). In contrast, formation of APLP1 high molecular weight complexes was unchanged between E14 and P30 and remained at a constant high level between 1 and 12 months of age (Fig. 6C, D).

Interestingly, the percentage of APLP2 dimerization in cortical mouse brain was strongest E14 and continuously decreasing until P30 (Fig. 6E). The signal remained constantly low in cortical mouse brains between 1 and 12 months of age (Fig. 6F).

Taken together, the relative abundance of APP dimers in mouse cortices peaked at P10/P12, the period of synapse formation and remained high at least until 12 months of age while APLP2 showed the opposite phenomenon with the highest amount of APLP2 dimers at E14, slowly decreasing and remaining constantly low until the age of 1 year. In contrast, the percentage of APLP1 high molecular complexes was constant during development and during adulthood.

### Dimer formation of different APP splice forms

**Fig. 7:**
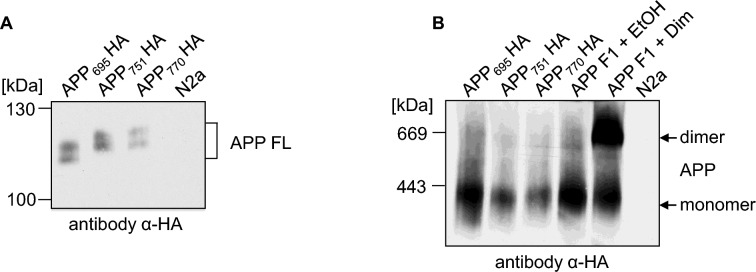
The dimer amount is independent of different APP splice variants. **A** APP_695_ HA, APP_751_ HA and APP_770_ HA were heterologously expressed in N2a cells. Non-transfected N2a cells served as a negative control. Equal amounts of cell lysates were separated on SDS-Gels and Western blot detection followed with an α-HA antibody. **B** APP_695_ HA, APP_751_ HA and APP_770_ HA or APP F1 were heterologously expressed in N2a cells. To show APP dimers, APP F1 expressing cells were treated with 100 nM of the rapamycin analogue AP20187 overnight to induce dimerization of APP. Incubation with the solvent of AP20187, ethanol, served as a negative control. The samples were analyzed via Blue Native PAGE and Western blot analysis followed with an α-HA antibody

Next, we wanted to address the question why dimerization of APP is strongly increased in mouse brain as compared to cell culture cells. For APP, we were analyzing so far splice form APP_695_ with the APP F1 construct. In general, eight different splice variants are known for APP whereby APP_695_, APP_751_, and APP_770_ are the three major isoforms being expressed, thereof APP_695_ primarily in neurons [39]. Therefore, we were testing if differences regarding the degree of APP dimerization are splice form dependent. We generated C-terminally HA tagged constructs of APP_695_, APP_751_, and APP_770_ and verified the correct sizes of these proteins after SDS gel analysis and Western blot detection with an α-HA antibody (Fig. 7A). Next, we compared APP_695_-HA, APP_751_-HA, and APP_770_-HA with the inducible APP_695_ F1 dimerization system via BN gel analysis. Western blot detection with an α-HA antibody revealed that APP dimer formation was comparable between the different APP splice forms suggesting that increased APP dimer formation in cortical mouse brains is independent of splice variants (Fig. 7B).

### APP family dimer formation in primary astrocytes and primary neurons

**Fig. 8:**
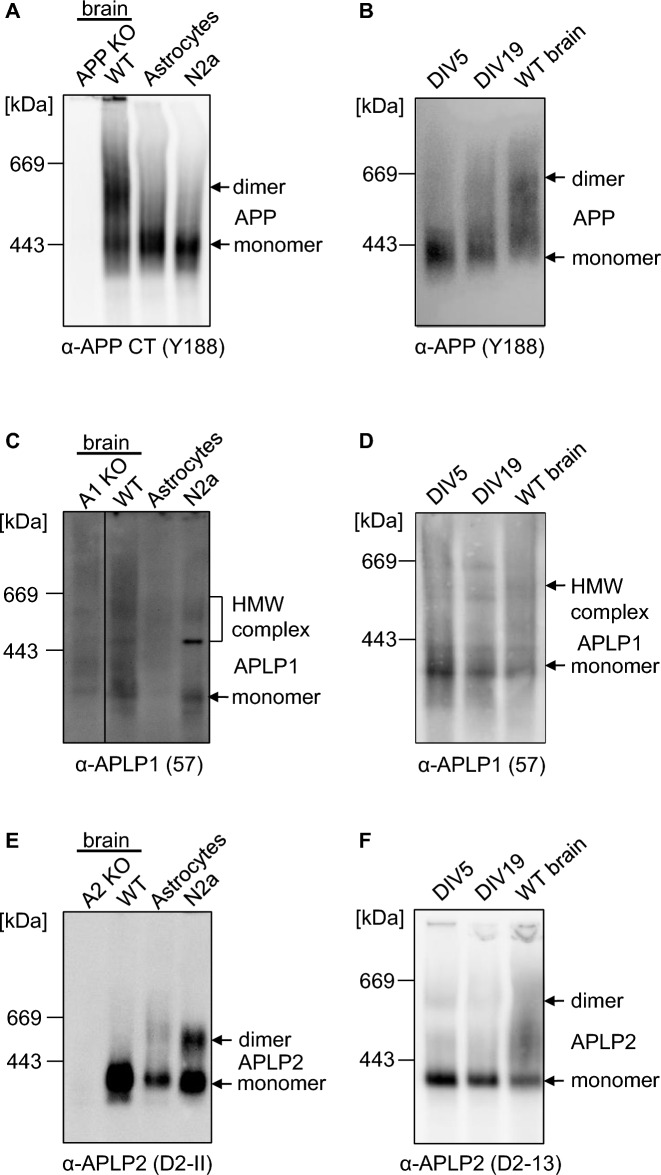
Higher amount of APP/APLP1/APLP2 dimerization is not visible in astrocytes, N2a cells or neurons. **A**, **C**, **E** One year old WT mouse cortices and APP KO mouse cortices were homogenized and compared with the lysate of astrocytes and N2a cells. All samples were prepared under semi-denaturing conditions for analysis on Blue Native gels. Western blot detection of APP followed with the primary α-APP C-terminal antibody Y188 (**A**), of APLP1 with the primary α-APLP1 antibody 57 (**C**) and APLP2 with the primary α-APLP2 antibody D2-II (**E**). **B**, **D**, **F** Mouse primary cortical neurons were cultivated for 5 or 19 days in vitro (DIV), lysed and compared to the lysate of 1 year old WT mouse cortices. All samples were prepared under semi-denaturing conditions for analysis on Blue Native gels. Western blot detection of APP followed with the primary α-APP C-terminal antibody Y188 (**B**), of APLP1 with the primary α-APLP1 antibody 57 (**D**) and APLP2 with the primary α-APLP2 antibody D2-II (**F**)

Next, we examined dimer formation of the APP family members in mouse cortical astrocytes, endogenously in mouse N2a cells and primary cortical neuronal cultures at an early stage DIV5 and a later stage of differentiation coinciding with synapse formation (DIV19) (Fig. 8). Firstly, we were analyzing the dimerization level of APP. APP dimers in cortical WT mouse brains showed again a percentage of ~ 45% while there were only very little APP dimers present in primary astrocytes (Fig. 8A) and primary cortical neuronal cultures (DIV5), but they were increased at DIV19 (Fig. 8B). APLP1 high molecular weight complexes in cortical WT mouse brains showed again a signal between 200 and 800 kDa, while there was no specific signal present for APLP1 in samples of cortical astrocytes, consistent with the previously shown neuron-specific expression of APLP1 [6] and a very low signal in mouse neuronal N2a cells (Fig. 8C). The APLP1 antibody used gives unfortunately rise to high background. APLP1 high molecular weight complexes between 200 and 800 kDa were also present in primary cortical neuronal cultures at DIV5 and DIV19 but did not reveal any differences (Fig. 8D). APLP2 dimers in cortical WT mouse brains showed again a low percentage while there were surprisingly more APLP2 dimers present in primary astrocytes and mouse neuronal N2a cells (Fig. 8E). Primary cortical neuronal cultures contained also only a very low amount of APLP2 dimers at DIV5 and DIV19 (Fig. 8F). Taken together, APP dimerization seems to be increased in primary neuronal cultures at the time point of synapse formation and APLP2 dimerization is increased in primary cultures of cortical astrocytes while no modulation was found for APLP1 in these different culture systems.

### APP family dimer formation in synaptosomes

**Fig. 9:**
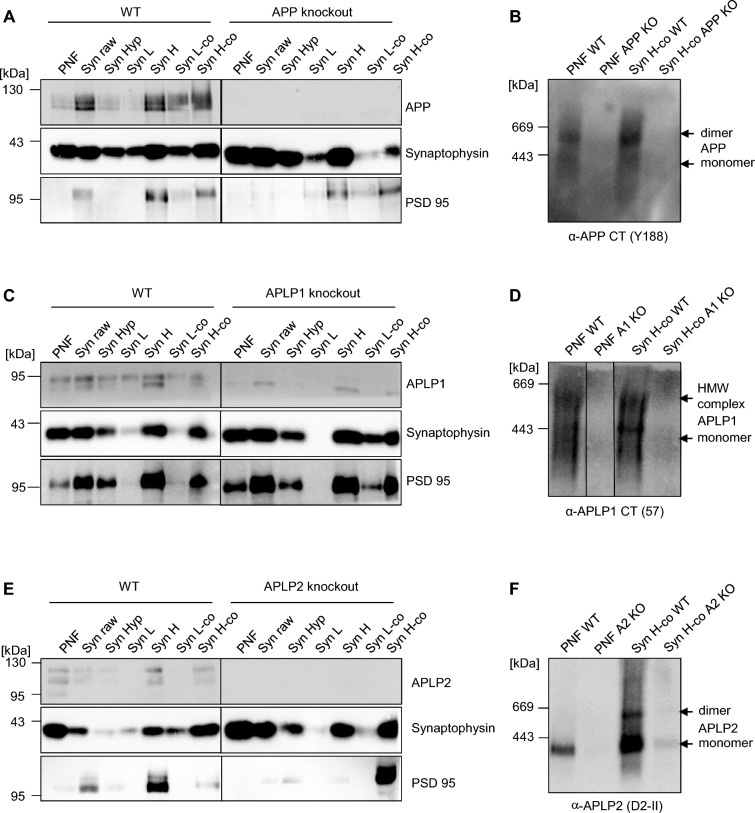
Expression analysis of APP, APLP1, and APLP2 dimers in mouse cortices in synaptosomal preparations. **A**, **C**, **E** WT mouse (C57BL/6) brain homogenates were sub fractionated by differential centrifugation steps and analyzed by Western blot with the primary α-APP C-terminal antibody Y188, α-APLP1 antibody 57, and α-APLP2 D2-II antibody. α-Synaptophysin and α-PSD-95 antibodies, respectively, were used as presynaptic and postsynaptic markers. To confirm specificity of the signals, synaptosome preparations were also performed with APP, APLP1, and APLP2 KO mouse brains. **B**, **D**, **F** Preparation of the post nuclear fraction (PNF) and the concentrated synaptosomes (Syn H-co) under semi-denaturing conditions for analysis of dimers on Blue Native gels. Western blot detection of APP followed with the primary α-APP C-terminal antibody Y188 (**B**), of APLP1 with the primary α-APLP1 antibody 57 (**D**) and APLP2 with the primary α-APLP2 antibody D2-II (**F**)

Via BN gel analyses, it can’t be distinguished if the visualized dimers form due to *cis-* or *trans*-dimerization. Based on the result of an increased presence of APP dimers in neuronal cultures at the time point of synapse formation, we were wondering if the higher rate of APP dimers in mouse brains is based on *trans*-dimerization at the synapse. Therefore, we were analyzing mouse brain synaptosomes using BN gels (Fig. 9). Synaptosomal fractions were prepared using WT and the corresponding cortical KO brains and examined via SDS PAGE and Western blot analysis. Synaptophysin and PSD 95 antibodies were used as a control for enrichment of pre- and postsynaptic parts of the synaptosomes, respectively (Fig. 9A, C, E). During this fractionation procedure, no TritonX-100 treatment to remove all presynaptic proteins was performed, since this strong detergent would have been disturbing the analysis of complexes on BN gels. BN gel analysis revealed an obvious increase in the amount of APP dimers in concentrated (co) synaptosomal fractions (Syn H-co), compared to post nuclear fractions (PNF) which contain crude cell lysates of the brain (Fig. 9B). The corresponding KO brain fractions were used as a control for specificity of the antibody signal. For APLP1, no major differences between the PNF and the Syn H-co fraction were observed (Fig. 9C) while for APLP2, similarly to APP, the signal of APLP2 dimers was increased in the Syn H-co fraction compared with the PNF fraction (Fig. 9F). Since we examined endogenous APLP2 in mouse brain in this experiment, we could theoretically have been analyzing here an isoform of APLP2 which can be modified via chondroitin sulfate glycosaminoglycan (CS GAG) attachment, which is APLP2-751 [40] or isoform APLP2-763 for which a 12-amino acid peptide is inserted two amino acids N-terminal to the CS GAG attachment site thereby preventing this modification [40].

In conclusion, our data indicate that APP and APLP2 dimerization may be elevated in synaptosomal fractions, possibly as a result of *trans*-dimerization occurring at the synapse.

### APP FAD mutations do not show an impact on APP dimerization

**Fig. 10:**
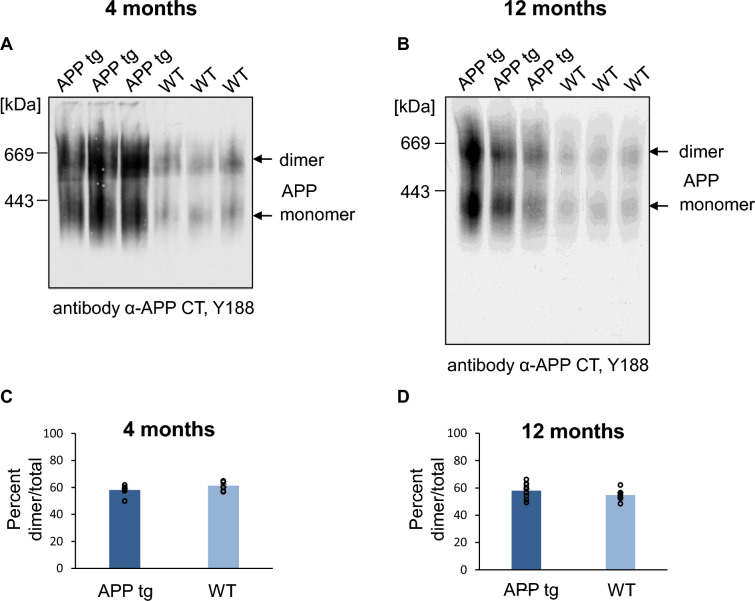
APP transgenic and WT mice show comparable amounts of APP dimer in the brain. **A**, **B** Mouse cortices of WT and of APP J20 transgenic mice at the age of 4 and 12 months. All samples were prepared under semi-denaturing conditions for analysis on Blue Native gels. Western blot detection of APP followed with the primary α-APP C-terminal antibody Y188. **C**, **D** Quantification of the data shown in A and B. Bars represent mean values ± SEM of dimer ratio to the total amount of the signal in %. *n* = 3, unpaired Student’s *t* test *p < 0.05, **p < 0.01, ***p < 0.001

We next examined if familial AD (FAD) mutations may impact APP dimer formation. Therefore, we analyzed cortical mouse brains of J20 mice, which are transgenic mice harboring alternatively spliced minigenes of hAPP_695_, hAPP_751_, and hAPP_770_ containing the APP FAD mutations Swedish (K670N/M671L) and Indiana (V717F) under a neuronal PDGF-β promotor [41–43]. J20 mice show the highest APP expression in the neocortex and hippocampus [44] and amyloid plaques are observed at 5–7 months in the dentate gyrus and neocortex in this AD mouse model [42, 44]. We investigated J20 cortical mouse brains for the presence of APP dimers at 4 months, before the onset of plaque formation and at 12 months when a high plaque load has developed. Non-transgenic littermates were used as a control. For the age of four months, we observed 58.14% ± 1.25% APP dimers in J20 mice and 61.31% ± 1.04% APP dimers in non-transgenic mice and for the age of 12 months 58.00% ± 1.3% APP dimers in J20 mice and 54.75% ± 1.8% APP dimers in non-transgenic mice. This indicates that there was no significant difference in the relative abundance of dimers in transgenic versus WT mice (Fig. 10A–D). The result of increased APP dimer formation in the J20 mouse line implies that the observed APP dimers most likely result from all three major splice forms of APP (APP_695_, APP_751_, and APP_770_). Furthermore, APP FAD mutations APP Swedish and APP Indiana do not seem to affect APP dimerization in mouse brains.

### APP dimerization in human brains

**Fig. 11:**
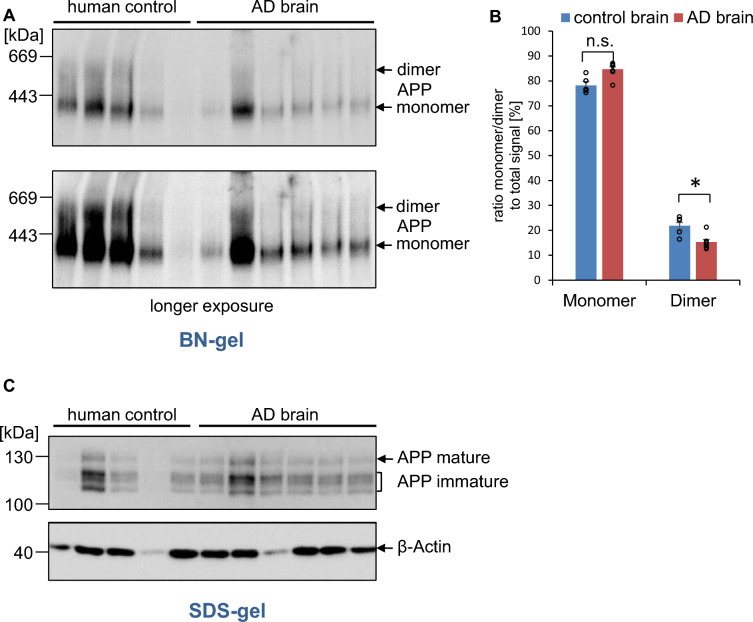
Alzheimer’s disease patients show decreased amounts of APP dimers in the brain. **A** Samples of the middle frontal gyrus of AD patients and human controls, age matched, and area matched, were lysed under semi-denaturing conditions for analysis on Blue Native gels. Western blot detection of APP followed with the primary α-APP C-terminal antibody Y188. **B** Quantification of data shown in A. Bars represent mean values ± SEM of monomer/dimer ratio to the total amount of the signal in %. n = 5 (human controls), n = 6 (AD brains), unpaired Student’s *t* test *p < 0.05, **p < 0.01, ***p < 0.001. **C** Samples of the middle frontal gyrus of AD patients and human controls, age matched, and area matched, were lysed in buffer containing 1% NP40 for analysis on SDS gels. Western blot detection of APP followed with the primary α-APP C-terminal antibody Y188 and an α-β-actin antibody was used to detect β-actin protein levels as a loading control

To address the question if APP dimerization plays a role during AD, age matched and area matched human AD brain samples of the middle frontal gyrus from sporadic AD patients were analyzed via BN gel analysis (Fig. 11, Table 1). Analysis of the same human brain samples via SDS gel and Western blot detection for fl. APP with α-Y188 antibody and visualization of β-actin are shown as a loading control (Fig. 11). Quantification revealed a significant decrease in the amount of APP dimers in brains of AD patients, suggesting that APP dimerization gets impaired during the course of AD.

**Table 1:** List of human brain samples

Number	Sex	Age	Pmd	pH	Diagnosis	Region
2010-062	Female	94	05:50	7.04	Human control	Middle frontal gyrus
2014-029	Female	78	07:10	6.32	Human control	Middle frontal gyrus
2012-086	Female	88	05:25	6.33	Human control	Middle frontal gyrus
2015-034	Female	82	07:45	5.97	Human control	Middle frontal gyrus
2012-033	Female	95	05:40	6.46	Human control	Middle frontal gyrus
2002-004	Female	91	04:15	6.27	AD brain	Middle frontal gyrus
2011-020	Female	89	04:00	6.40	AD brain	Middle frontal gyrus
2000-062	Female	91	03:45	6.36	AD brain	Middle frontal gyrus
2006-006	Female	87	05:00	6.37	AD brain	Middle frontal gyrus
2004-030	Female	89	04:40	6.28	AD brain	Middle frontal gyrus
2001-120	Female	87	04:00	6.61	AD brain	Middle frontal gyrus

*Pmd* postmortem delay

## Discussion

### Comparison of APP, APLP1, and APLP2 complexes

APP, APLP1, and APLP2 can form dimers in *cis*- as well as in *trans-*orientation predominantly via the E1 domain in the extracellular domain [26–28]. On the other hand, the E2 domain has been found to dimerize as an antiparallel dimer suggesting that this domain might be involved in *trans*-dimerization of APP as well [45].

The degree of APP dimerization in cell culture cells has already been investigated using BN gel analyses and an APP-FKBP fusion protein [29]. With this system, after addition of the dimerization agent AP20187, ~ 66% APP dimers could be induced in N2a cells, while only about 22% APP dimers were present in the control (Figs. 1, 5) [29]. This suggests that approximately 22% of APP molecules are dimerized in cells, cultivated in vitro, which is in line with other independent reports [46, 47]. Furthermore, cross-linking studies in human SH-SY5Y cells corroborate the low proportion of APP *cis*-dimers in cell culture cells [26, 36] and also FRET experiments indicated APP homo-dimerization to a degree of 11% in HEK cells [28, 37].

Together, our data suggest that APP dimers represent only a minor proportion of total APP in cells, cultivated in vitro in cell lines, which are not forming synapses. The amount of *trans*-dimers is assumed to be even lower under these conditions. Cell surface localization of proteins is the prerequisite for their interaction in *trans*-orientation. Therefore, studies showing a low amount of APP at the cell surface suggest that only a low proportion of APP dimerizes in *trans*-orientation [30]. The pattern of complexes on BN gels obtained from transiently transfected N2a cells was quite comparable between APP and APLP2 showing the majority of the signal to be in the monomeric form (Figs. 1, 2). In contrast, APLP1 revealed a pattern of steady complex formation between 200 and 800 kDa, involving also dimerized complexes (Figs. 1, 2). The reason for the pattern of diverse high molecular weight complexes might be based on heparin or zinc induced APLP1 dimerization [48–52] or differently glycosylated complexes [15, 53]. Furthermore, the different appearance of high molecular weight complexes of APLP1 might be based on different interaction partners of APLP1 compared to APP or APLP2, which has also been shown in earlier studies via a proteomic analysis of APP, APLP1, and APLP2 binding partner in mouse brain [54]. In addition, processing by e.g. the transmembrane serine protease Matriptase, which cleaves APLP1 within the E1 domain and thereby negatively impacts APLP1 homodimerization may contribute to the complex pattern of APLP1 on BN gels [55]. We already demonstrated that all APP family members can heterodimerize with each other in all possible combinations, which has also been shown endogenously in mouse brains via co-immunoprecipitation of APP and APLP1 in synaptosomes [26] (Additional file 1: Fig. S1). Therefore, the signals observed for endogenous APP, APLP1 or APLP2 complexes in mouse brains can also partially imply heterocomplexes of the APP family members.

Our results fit to the observation that APP and APLP2 share more properties in comparison to APLP1, which has several unique features. APLP1 is exclusively present in neurons [6] while APP and APLP2 are expressed ubiquitously [56]. For APLP1, only one isoform has been reported [53, 57], whereas alternative splicing leads to four mRNA variants for APLP2 [58] and eight splice variants for APP [59]. Also, a higher presence for APLP1 compared with APP or APLP2 at the cell surface has been reported via cell surface biotinylation [15] and ICC [28] which we confirmed in our study (Fig. 2). Additionally, we could demonstrate that deleting the C-terminus of APP or APLP2 including their internalization motifs, shifts their presence to the cell surface, resulting in a higher amount of *trans*-dimers in eukaryotic cell lines in the case of APP (Fig. 2A, B). Regarding *trans*-dimerization of the APP family members, we previously showed in vitro that APLP1 has the strongest *trans*-interacting properties and APP revealed the lowest degree of *trans*-dimerization [15], which is in line with APLP1 having the strongest impact of the APP family members on presynaptic differentiation, presumably via *trans*-interaction with APLP1 in the presence of APLP2 [15].

Together, we observed in heterologously expressing N2a cells, a relatively low amount of APLP2 and APP homotypic dimers while APLP1 showed a clearly higher degree of complex formation. Notably, other studies, using FRET analysis of transiently transfected HEK cells expressing APLP1 and APLP2 homo-dimers, reported that about 16% of total APLP1 and APLP2 are dimerized [28]. However, FRET analysis only allows detection of two fusion proteins (e.g. APLP1 fused to CFP and YFP) in close proximity, but does not test for direct binding. The low amount of detected APLP1 dimerization via FRET in contrast to our results might be based on conformational differences of the APLP1 fusion proteins or might be affected by the length of the linker region. Our co-immunoprecipitation data as well as BN gel analyses indicate strong dimerization of APLP1 (Figs. 1, 2, 3, 5).

### Diversity of APP, APLP1, and APLP2 complex formation in mouse brains

Strikingly, we observed for APP a clearly elevated amount of high molecular weight complexes in brain homogenates compared to cell culture cells (Fig. 5). In contrast, the degree of APLP1 and APLP2 dimerization, although very different, did not vary between cell culture and mouse brain homogenates (Fig. 5). Also, developmental alterations in APP, APLP1, and APLP2 complex formation differed between the APP family members. APP complex formation was mostly increased during synapse formation when APLP2 complex formation was low, and vice versa (Fig. 6), suggesting that APP, APLP1, and APLP2 dimerization are underlying a regulation that depends on developmental time point and cell type (Fig. 8).

In this work, we were able to demonstrate that APP complex formation is increased in mouse cortices compared to cell culture cells from 22 to ~ 49% (Fig. 5). The signals for APP monomers and dimers in mouse brain have been identified at ~ 300 kDa and ~ 600 kDa by comparison with dimerized and non-dimerized APP FKBP in N2a cells (Fig. 5). In contrast, APP monomers and APP dimers covalently induced by cysteine mutants APP L17C and APP K28C show an apparent molecular weight of ~ 120 kDa and ~ 240 kDa after separation in SDS gels [30] suggesting that APP is complexed with one or more additional binding partner, which is visible after BN gel analysis.

The increased occurrence of APP dimers in mouse cortices compared to cell culture cells could be related to more *trans*-dimers being formed in intact tissue compared with cell culture cells. In cell culture, cell–cell contacts are built at higher confluency for a short period of time during cultivation and due to increased presence at the cell surface (Fig. 4). One reason for this might be APP–APP *trans*-interaction at the synapse. Consistently, APP was shown to localize at both, the pre- and postsynapse [15, 60], which is the basic requirement for binding in *trans*-orientation at the synapse. Furthermore, synaptic *trans*-dimerization of APP proteins has been reported to promote synapse formation [15, 17, 26, 34]. Moreover, this assumption is supported by our data, showing that APP dimerization in mice has a peak at P10/P12, the period of synapse formation in mouse brains (Fig. 6) and that the level of APP dimers appeared to be increased in synaptosomes (Fig. 9).

Several factors have been described to influence APP dimer formation, such as Sortilin-Related Receptor Containing LDLR A Repeats (SorLA). SorLA is a protein that controls processing and intracellular transport of APP and impairs the formation of APP dimers in the brain [33]. SorLA KO mice show significantly higher amounts of APP dimers with a simultaneously strong reduction in APP monomers [33] suggesting that SorLA promotes the formation of APP monomers and inhibits APP dimerization.

Furthermore, copper induces dimer formation of APP in both, *cis*- as well as *trans*-orientation [50, 61] and also Cholesterol lowering drugs seem to promote APP dimerization [62]. Another factor inducing APP dimerization is the glycosaminoglycan heparin [63, 64]. It has been reported that APP seems to be almost completely dimerized at the cell surface and that this involves the presence of heparan sulfate at the plasma membrane [65]. Surface localization of APP is a balance resulting from its transport in the secretory path to the plasma membrane and its removal by either internalization or processing [30, 38, 66, 67]. For example, reduced proteolysis of APP in processing deficient mutants leads to an increased surface localization of APP, resulting in elevated *trans*-dimerization and synapse formation [34]. APP internalization from the cell surface occurs predominantly by Clathrin-mediated endocytosis via the NPTY motif in the C-terminus of APP [38, 68], but also via the basolateral Sorting sequence (BaSS) of APP which is the YTSI motif C-terminal of the transmembrane domain [38, 69].

As a type I transmembrane protein, APP structurally resembles a cell surface receptor [70], a property shared by the close homologues APLP1 and APLP2. It has been discussed that localization of APP at the plasma membrane allows the interaction with other proteins such as the APLPs, which is mediated by the N-terminal E1 domain [71]. The APP ectodomain has been reported to adopt different conformations depending on cellular pH [72]. A pH-dependent interaction between the GFLD subdomain with the copper-binding domain (CuBD) within the E1 domain might lead to a closed conformation for APP in a more acidic compartment like endosomes, whereas it will adopt a more open conformation at neutral pH 7.4 at the cell surface [72]. Therefore, APP in its open conformation at the plasma membrane can expose the E1 domain to allow association with other receptors or ligands resulting in different physiological functions. This is consistent with noticeable high molecular weight complex formation of the APP family members after BN gel electrophoresis (Figs. 1, 2, 5). The APP protein has only a very short half-life time of about 45 min [73, 74] implicating only a brief dwell time of APP at the plasma membrane. This indicates that *trans*-dimerization of APP, homophilic or heterophilic, is most likely not essential for stabilization of cell–cell interactions but will rather induce signal transduction processes. One interesting APP interaction partner is the protein N-cadherin, which is involved in the initiation of synapse formation [75] reported to increase APP dimerization in *cis*-orientation [76].

It was shown that *cis*-homo-dimerization of APP and/or the β-C-terminal fragment significantly reduced the amount of total Aβ [29, 62, 77, 78]. This suggests that a higher proportion of APP monomers leads to increased Aβ generation, which in turn should lead to more Aβ oligomers and ultimately more Aβ plaques. Thus, higher amounts of APP dimers might have a long-term positive effect in AD patients. In this work, it could be shown that APP in human cortex samples from non-demented controls is present at 21.8 ± 1.3% as a dimer and 78.2 ± 1.3% as a monomer (Fig. 11). Interestingly, the proportion of APP dimers is significantly reduced in samples from AD patients (Fig. 11). The amount of APP dimers is lower in human brain compared to mouse brain. We suspect that the delay in freezing human brain is the main reason for this discrepancy. The postmortem delay time for the analyzed human brains has been indicated with 4–7.45 h (Table 1). In contrast, all mouse brains used in this study have been shock frozen in liquid nitrogen immediately after cervical dislocation and preparation of the cortex using a standardized procedure which takes ~ 10 min per brain. In addition, there might be differences in the degree of APP dimerization in different brain regions. We were analyzing the middle frontal gyrus (grey matter) of human AD and control brains while we have been examining the entire cortex of mouse brains which includes also white matter. It would be very interesting to analyze the amount of APP dimers in white matter samples of human AD brain in future studies.

## Conclusions

Together, this could indicate that APP *trans*-dimerization, for example at synaptic adhesion sites, is severely reduced in brains of AD patients. Therefore, factors influencing this interaction play an important role in AD. This is also consistent with loss of synapses which occurs as an early feature of AD pathogenesis [79]. Thus, pharmacological approaches targeting APP dimerization properties might open novel strategies for treatment of AD.

## Methods

### Plasmids

The following plasmids were used: generation of APP F1 pC4 F1 has been described [29]. For cloning of APLP1 pC4 F1, the vector pC4 F1 (Clontech) has been digested EcoRI–XbaI. APLP1 WT in pBluescript SK+ was digested with EcoRI and BamHI to obtain a 1358 bp fragment. A PCR was performed to add an XbaI site at the 3′ end of the APLP1 ORF. APLP1 WT in pBluescript Sk+ served as a template [53]. The sense primer starts 30–50 bp before the BamHI site in the APLP1 ORF 5′ GAGCAGAAGGAACAGAGGCA 3′ and the antisense primer including XbaI was as follows 5′ GTCAGTTCTAGAGGGTCGTTCCTCCAGGAAG 3′. The PCR product was digested BamHI and XbaI and ligated with the EcoRI–BamHI fragment via EcoRI and XbaI in vector pC4 F1. APLP2 pC4 F1 was cloned by cutting dimerization vector pC4 F1 (Clontech) EcoRI–XbaI. APLP2-763 WT in pCEP4 [3] was digested EcoRI–XhoI to obtain a 2100 bp fragment. A PCR was performed using sense primer 5′ C ATG GTC ATT GAC GAG ACT C 3′ starting ~ 30 bp before the internal unique XhoI site of APLP2-763 ORF. The following antisense primer 5′ GTCAGCTCTAGAAATCTGCATCTGCTCCAGG 3′ was used to append an XbaI site to the 3′ end of the APLP2 ORF. The PCR product was cut XhoI–XbaI. The EcoRI–XhoI fragment and the XhoI–XbaI fragment were ligated EcoRI–XbaI in dimerization vector pC4 F1.

Generation of APLP1 CT HA pcDNA3.1+ neo has been described [15]. Cysteine dimerization mutants APLP1 R579C CT HA pcDNA3.1+ neo, APLP1 E580C CT HA pcDNA3.1+ neo were cloned via site directed mutagenesis and verified by sequencing.

Cloning of APLP2 CT HA pcDNA3.1+ neo has been described [26]. Cysteine dimerization mutants APLP2 CT HA L690C pcDNA3.1+ neo, APLP2 CT HA S692C pcDNA3.1+ neo were obtained via site directed mutagenesis and verified by sequencing.

Generation of APP695 CT HA pcDNA3.1+ neo has been described [29]. To obtain APP751 CT HA pcDNA3.1+ neo, an HA tag has been appended to the C-terminus of APP751 WT pcDNA3.1+ neo via PCR. Sense primer 5′ GAAGTTGAGCCTGTTGATGCC 3′ starts at position 1848 in the hAPP751 ORF. Antisense primer 5′ GCTGACCTCGAGTTATGCGTAGCTGGTACGTCGTACGGATAGTTCTGCATCTGCTC 3′ contains an XhoI site and an HA tag. The 400 bp PCR product was cut BglII–XhoI to obtain a 350 bp fragment. APP751 WT pcDNA3.1 was digested EcoRI–BglII to obtain the remaining insert of 1940 bp. The vector pcDNA3.1+ neo containing the 3′ UTR of APP [29] was cut EcoRI and XhoI to ligate in the EcoRI–BglII and BglII–XhoI fragments.

APP770 CT HA pcDNA3.1+ neo was generated by appending a C-terminal HA tag to APP770 WT in pcDNA3.1+ neo via PCR. Therefore, the following sense primer was used 5′ GAAGTTGAGCCTGTTGATGCC 3′ starting at position 1906 in the hAPP770 ORF and an antisense primer 5′ GCTGACCTCGAGTTATGCGTAGTCTGGTACGTCGTACGGATAGTTCTGCATCTGCTC 3′ including an XhoI site and an HA tag. The resulting PCR product: was digested BglII–XhoI to obtain a 350 bp fragment. APP751 WT in pcDNA3.1+ was cut EcoRI–BglII to obtain the remaining insert of 1994 bp. Vector 3′ UTR APP in pcDNA3.1+ neo [29] was digested EcoRI and XhoI. The EcoRI–BglII and BglII–XhoI fragments were ligated in the EcoRI–XhoI digested vector.

Generation of the plasmids Myc APP∆CT 648 pcDNA3.1+ neo and HA APP∆CT 648 pcDNA3.1+ neo was based on site directed mutagenesis by introducing a stop codon after position 648 at the C-terminal end of the APP transmembrane domain of spliceform APP695. The following primer pair was used: 5′ C ACC TTG GTG ATG CTG TGA AAG AAG AAA CAG TAC 3′ and 5′ GTA CTG TTT CTT CTT TCA CAG CAT CAC CAA GGT G 3′. N-terminally c-myc tagged APLP1∆CT and c-myc tagged APLP2∆CT constructs have already been described [26]. N-terminally HA tagged APLP1∆CT pcDNA3.1+ neo and N-terminally HA APLP2∆CT pcDNA3.1+ neo constructs have been generated by introducing a stop codon C-terminal to the transmembrane domain of APLP1, or APLP2, respectively.

### Antibodies

Primary rat monoclonal antibodies included α-HA antibody (000000011867431001, 3F10, Roche, Rotkreuz, Switzerland). Primary mouse monoclonal antibodies α-c-myc antibody (9E10) (Ab32, Abcam, Cambridge, UK), PSD-95 (Abcam, Cambridge, UK), Synaptophysin (Sigma-Aldrich), Golgi Marker GM130 (BD Biosciences). Further, the following rabbit polyclonal antibodies were used: α-c-myc (A-14, sc-769, Santa Cruz, Nunningen, Switzerland), Y188 (APP C-terminal antibody, Epitomics), α-APLP1 (57; [3]), D2-II, APLP2 C-terminal antibody (Calbiochem), which is not commercially available anymore.

### Cell lines and transfections

N2a mouse neuroblastoma cells were maintained in the following medium: Minimum Essential Medium (MEM, Gibco) supplemented with 10% FBS (Sigma), 1% penicillin/streptomycin (Gibco), 1% l-glutamine (200 mM) (Sigma), 1% non-essential amino acids, 1% sodium pyruvate. HeLa Kyoto cells were maintained in the following media: Dulbecco’s modified Eagle’s medium (DMEM, Gibco) supplemented with 10% FBS (Sigma), 1% l-glutamine (200 mM) (Sigma), and 1% penicillin/streptomycin (Gibco). For Western blot analysis, cells were transfected with jetPRIME (Polyplus, Illkirch, France) according to manufacturer’s instructions. Dimerizer AP20187 (B/B) (Clontech, Saint-Germain-en-Laye France) was added 4 h post-transfection overnight.

### Mice

Generation and genotyping of knock-out lines were described previously for APP KO and APLP1 KO [80] as well as APLP2 KO mice [81]. All mice have been backcrossed at least six times to C57BL/6 mice. Sex of the species used is of either sex. C57BL/6J mice [embryonic day 14 (E14)] were used for the generation of primary cortical neuron cultures. Transgenic J20 mice express APP Indiana (V717F) and Swedish (K670M) mutations on a C57BL/6 background [42]. Mice were treated in accordance with the German law for conducting animal experiments and followed the National Institutes of Health *Guide for the Care and Use of Laboratory Animals*. Animal housing, breeding, and the sacrifice of mice were approved by the German administration. All experimental protocols were performed in accordance with the European Communities Council Directive of 24 November 1986 (86/609/EEC).

### Blue native gel analysis

Blue Native gel electrophoresis was performed according to a protocol modified from [82].

#### For analysis of transiently transfected N2a cells

In brief, cells in one 10 cm cell culture dish were washed once and collected in phosphate-buffered saline at 4 °C. The cell pellets were resuspended in 1 ml of homogenization buffer (250 mM sucrose in 20 mM HEPES, pH 7.4, with protease inhibitor mix “Complete”, Roche Rotkreuz, Switzerland) and then sheared by passing through a 27× gauge needle 10 times.

#### For analysis of mouse brains

Mouse cortices were homogenized in homogenization buffer (250 mM sucrose in 20 mM HEPES, pH 7.4, with protease inhibitor mix “Complete”, Roche Rotkreuz, Switzerland) at 800 rpm 13 times with a glass-Teflon homogenizer on ice.

The following steps are identical for cells as well mouse cortices. Post nuclear supernatant was collected after a low-speed spin at 300×*g* for 15 min at 4 °C. Membranes were pelleted after centrifugation at 100,000×*g* for 1 h at 4 °C and washed once with 200 µl of homogenization buffer. After repeating the ultracentrifugation step, pellets containing the membranes were resuspended in 200 µl homogenization buffer.

50 µg protein was solubilized with Blue Native sample buffer (1.5 M amino caproic acid, 0.05 M Bis–Tris, 10% *n*-dodecyl-β-d-maltoside, and protease inhibitor at pH 7.5). The samples were incubated on ice for 30 min and then centrifuged for 10 min at 14,000 rpm at 4 °C in a microcentrifuge. Blue Native loading buffer (5.0% Serva Coomassie Brilliant Blue G250 and 1.0 M aminocaproic acid) was added to the supernatant. The samples were separated on 4–15% Tris–HCl gels (Criterion, Bio-Rad) overnight at 4 °C with Coomassie Blue containing cathode buffer (10× cathode buffer, pH 7.0, 0.5 M Tricine, 0.15 M Bis–Tris, 0.2% Coomassie Blue) and anode buffer (pH 7.0, 0.5 M Bis–Tris). The gel was transferred to a polyvinylidene difluoride membrane. The following molecular weight standards were used: thyroglobulin (669 kDa), apoferritin (443 kDa), catalase (240 kDa), aldolase (158 kDa), and bovine serum albumin (66 kDa), all from Sigma-Aldrich, Munich, Germany.

### *Cis*-co-immunoprecipitation

*Cis*-co-immunoprecipitations were performed, as described before [30]. Briefly, N2a cells co-expressing APP CT HA and APP CT myc, APLP1 CT HA and APLP1 CT myc or APLP2 CT HA and APLP2 CT myc were harvested and lysed in 50 mM Tris/HCl pH 7.5; 150 mM NaCl; 5 mM EDTA; 1% NP40; 1:25 protease inhibitor (Complete (with EDTA), Roche, Rotkreuz, Switzerland) 18 h post-transfection. Equal volumes of cell lysates containing ~ 1000 μg protein were precleared with 10 μl Protein A Sepharose (GE Healthcare, Freiburg, Germany) for 1 h at 4 °C. Afterwards, the supernatant was incubated at 4 °C overnight with 20 μl α-HA antibody coated beads (Roche, Rotkreuz, Switzerland) at RT. After several washing steps with lysis buffer and 10 mM Tris/HCl pH 7.5, the beads were resuspended in SDS sample buffer (0.125 M Tris/HCl pH 6.8; 20% glycerol; 4% SDS; 0.01% bromophenol blue; 100 mM DTT) and incubated for 5 min at 95 °C. The samples were loaded on an 8% Tris/glycine gel and subjected to Western blot analysis using different primary antibodies [α-c-myc (A-14), sc-769, Santa Cruz, Nunningen, Switzerland and α-HA antibody, rat, 3F10, Sigma-Aldrich, Munich, Germany].

### *Trans*-co-immunoprecipitation

*Trans*-co-immunoprecipitations were performed by transiently transfecting N2a cells (10 cm dishes) using JetPrime (Polyplus) expressing APP CT HA, APP CT myc, APLP1 CT HA, APLP1 CT myc, APLP2 CT HA or APLP2 CT myc. 4 h after the transfection, the following cells of two dishes were combined in one 6 cm dish: APP CT HA and APP CT myc, APLP1 HA and APLP1 CT myc, APLP2 HA and APLP2 CT myc expressing cells. 18 h post transfection, the cells were harvested and lysed in 50 mM Tris/HCl pH 7.5; 150 mM NaCl; 5 mM EDTA; 1% NP40; 1:25 protease inhibitor (Complete (with EDTA), Roche, Rotkreuz, Switzerland). Further steps followed as described above for *cis*-co-immunoprecipitations.

### Sample preparation for SDS Gels and Western blot

Cells were lysed for 15 min at 4 °C in lysis buffer (50 mM Tris/HCl, pH 7.5, 150 mM NaCl, 5 mM EDTA, and 1% Nonidet P40) supplemented with protease inhibitors (CompleteTM protease inhibitor mixture, Roche, Rotkreuz, Switzerland). Supernatants were collected, and the protein concentration was determined with a BCA assay (Sigma, Deisenhofen, Germany). Equal amounts of protein samples were separated on 8% Tris/glycine gels and then transferred to nitrocellulose membranes (GE Healthcare, Uppsala, Sweden). Subsequently, the membranes were incubated with primary antibodies and HRP-coupled secondary antibodies (Jackson ImmunoResearch, West Grove, Pennsylvania, USA). Chemiluminescence was measured, using an imager and the software Fusion (Vilber Loumat).

### Synaptosomal preparation and enrichment of the postsynaptic density

Samples were always kept on ice, and all centrifugation steps were performed at 4 °C. One mouse brain was homogenized in solution A (0.32 M sucrose; 1 mM NaHCO_3_; 1 mM MgCl_2_; 0.5 mM CaCl_2_) by a Potter S Homogenizer. Centrifugation for 10 min at 800×*g* sedimented crude cell fragments [“post-nuclear supernatant” (PNF)]. The supernatant was centrifuged for 15 min at 9000×*g* (smaller cell components stay in the supernatant). The pellet was resuspended in solution A, centrifuged again for 15 min at 10,000×*g*, and resuspended in solution B (0.32 M sucrose; 1 mM NaHCO_3_) to obtain a raw synaptosomal fraction (Syn raw). Synaptosomal membranes were isolated via hypo osmotic shock by the addition of double-distilled water. The reaction was stopped with 0.5 M HEPES/NaOH, pH 7.4 [fraction “synaptosomes after hypoosmotic shock” (Syn Hyp)]. The solution was centrifuged for 20 min at 25,000×*g*, and the pellet was subsequently (dissolved in solution B) loaded on a sucrose gradient (0.5 M–1 M–2 M sucrose in 1 mM NaHCO_3_). A discontinuous density centrifugation at 82,500×*g* was performed for 3 h. The low dense fraction (Syn L) and the high dense fraction were collected (Syn HdFr) and centrifuged again at 201,800×*g* for 20 min. Pellets were subsequently resuspended in 5 mM Tris/HCl (PSD L and PSD H). To analyze equal amounts of proteins, a BCA test was performed, and the samples were loaded on an 8% Tris/glycine gel or a Blue Native gel.

### Immunocytochemistry

HeLa cells were seeded at a density of 35,000 cells per well in a 24-well plate (Greiner) on 14-mm coverslips and transfected via jetPrime. The cells were fixed after 18–20 h for 10 min at 37 °C in 4% PFA with 4% sucrose and permeabilized for 10 min with 0.1% NP40. After incubation of primary antibodies at 4 °C overnight and secondary antibodies for 1 h at RT (Alexa Flour 488 and 594) cells were embedded in Mowiol (Sigma-Aldrich) and subjected to imaging with the software Axiovision 4.8 at the microscope Axio Observer Z.1.

### Cell surface staining

HeLa cells were seeded at a density of 35,000 cells per well in a 24-well plate (Greiner) on 14-mm coverslips and transfected via jetPrime. After 18–20 h, the cells were cooled on ice and incubated with an α-c-myc antibody to stain only proteins which were localized at the surface. Subsequently, the cells were fixed for 10 min on ice in 4% PFA with 4% sucrose and another 20 min at RT. As a next step, they were incubated with the secondary antibody Alexa Flour 488 for 1 h at RT. After washing, the cells were permeabilized for 10 min with 0.1% NP40 and stained again with the same α-c-myc antibody to also visualize intracellular proteins. The secondary antibody Alexa Flour 594 was again added for 1 h at RT. After several washing steps, the cells were embedded in Mowiol (Sigma-Aldrich) and subjected to imaging with the software Axiovision 4.8 at the microscope Axio Observer Z.1.

## Supplementary Information

**Additional file 1: Figure S1.** APP forms heterotypic *trans* dimers with APLP1 and APLP2 including post lysis mixture control.

## Data Availability

The datasets generated during and/or analyzed during the current study are available from the corresponding author on reasonable request.
