# Supplementary material for: APP family member dimeric complexes are formed predominantly in synaptic compartments
Source: Cell Biosci. 2023 Aug 2;13:141. doi: 10.1186/s13578-023-01092-6 (PMC10398996; doi:10.1186/s13578-023-01092-6)
Supplement: Supplementary file 1 — Additional file 1: Figure S1. APP forms heterotypic trans dimers with APLP1 and APLP2 including post lysis mixture control. [file 13578_2023_1092_MOESM1_ESM.pdf]

Figure S 1

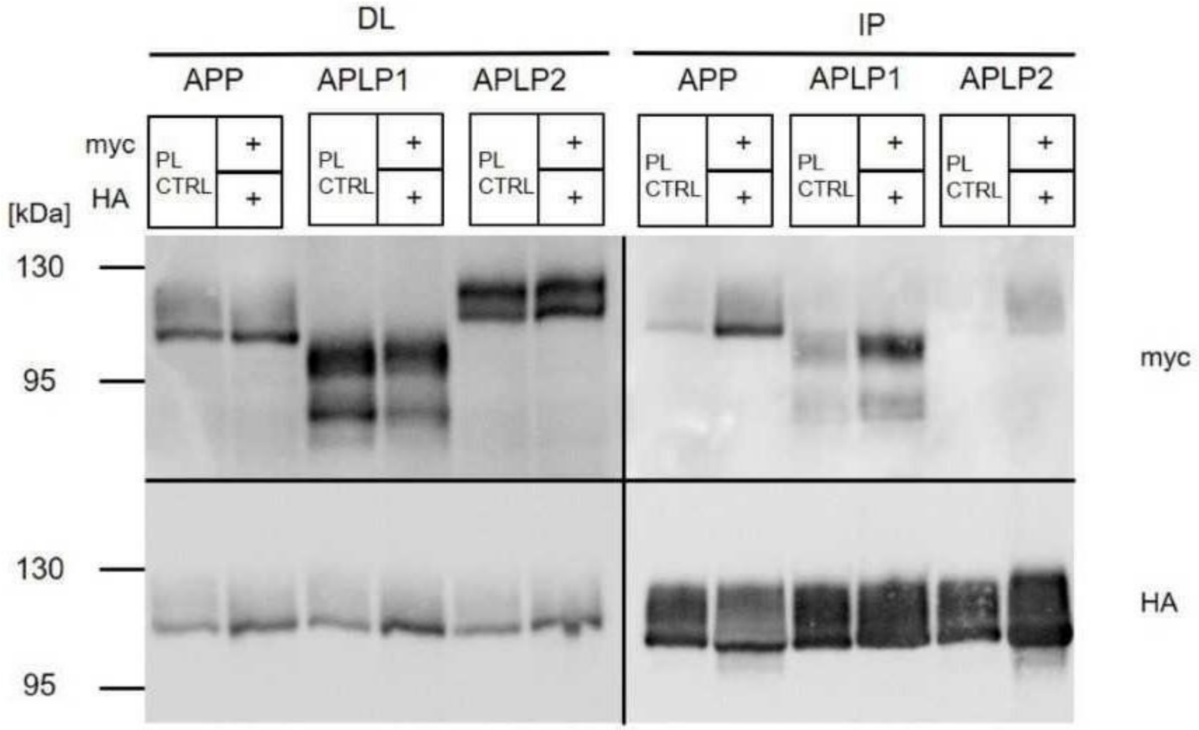

Figure S1. APP forms heterotypic *trans* dimers with APLP1 and APLP2 including post lysis mixture control

HA-tagged APP and myc-tagged APP, APLP1 or APLP2 were expressed in HEK cells and pairs of myc- and HA-tagged proteins were co-cultivated overnight (HA-APP and myc-APP; HA-APP and myc-APLP1; HA-APP and myc-APLP2).

Separately transfected HA-APP and myc-APP, -APLP1 or APLP2 cells were mixed post-lysis and used as a control ("PL CTRL"). 20 ug of each lysate was used as an input control (DL) whereas 1000 ug of each protein lysate was used for immunoprecipitation (IP) with HA-coupled beads. The samples were separated on an 8% Tris/glycine gel, electrophoresed and analyzed by Western blot. The primary antibody used to detect myc-tagged APP, APLP, and APLP2 proteins was the anti-myc antibody 9E10. The post-lysis mixture control confirms specificity of the interaction.
